# Electronic Cigarette Vaping with Nicotine Causes Increased Thrombogenicity and Impaired Microvascular Function in Healthy Volunteers: A Randomised Clinical Trial

**DOI:** 10.1007/s12012-023-09802-9

**Published:** 2023-08-07

**Authors:** Gustaf Lyytinen, Amelie Brynedal, Erik Anesäter, Lukasz Antoniewicz, Anders Blomberg, Håkan Wallén, Jenny A. Bosson, Linnea Hedman, Fariborz Mobarrez, Sara Tehrani, Magnus Lundbäck

**Affiliations:** 1 Department of Clinical Sciences, Division of Cardiovascular Medicine, Karolinska Institutet, Danderyd University Hospital, Stockholm, Sweden; 2 Section of Medicine, Department of Public Health and Clinical Medicine, Umeå University, Umeå, Sweden; 3 Department of Medicine II, Division of Pulmonology, Medical University of Vienna, Vienna, Austria; 4 Section of Sustainable Health, Department of Public Health and Clinical Medicine, The OLIN Unit, Umeå University, Umeå, Sweden; 5 Department of Medical Sciences, Uppsala University, Uppsala, Sweden; 6 Department of Clinical Sciences, Division of Internal Medicine, Danderyd University Hospital, Karolinska Institutet, Stockholm, Sweden

**Keywords:** Electronic cigarette, Nicotine, Thrombosis, Microcirculation, Platelets, Coagulation, E-cigarette

## Abstract

Electronic cigarette (EC) vaping is increasingly popular, despite growing evidence of adverse health effects. To further evaluate the impact of EC use on vascular health, we investigated the effects of brief EC inhalation on flow-dependent thrombus formation and microcirculation in healthy volunteers. The study was performed with a randomised double-blind crossover design. Twenty-two healthy subjects aged between 18 and 45 years with occasional tobacco use were recruited. Subjects inhaled 30 puffs of EC aerosol with and without nicotine on two occasions separated by a wash-out period of at least 1 week. Blood samples were collected at baseline and at 15 and 60 min following exposure and analysed with the Total-Thrombus-formation analysis system evaluating fibrin-rich thrombus formation and platelet thrombus formation in whole blood under flow. Microvascular function was assessed at baseline and 30 min after exposure by laser speckle contrast imaging and iontophoresis of acetylcholine and sodium nitroprusside (SNP) to evaluate the endothelium-dependent and independent pathways of vasodilation. Compared with nicotine free EC aerosol, exposure to EC aerosol with nicotine significantly increased platelet thrombus formation and fibrin-rich thrombus formation at 15 min (*p* = 0.017 and *p* = 0.037, respectively) with normalisation after 60 min. Peak SNP-mediated microvascular perfusion, i.e. endothelium-independent vasodilation, was reduced following EC vaping with nicotine compared with baseline (*p* = 0.006). Thirty puffs of EC aerosol with nicotine increased platelet and fibrin-dependent thrombus formation and reduced microvascular dilatation capacity. No compelling effects of EC vaping without nicotine were observed, indicating nicotine as the main effector.

**Trial registration**: ClinicalTrials.gov Identifier: NCT04175457 URL: https://clinicaltrials.gov/ct2/show/NCT04175457

**Supplementary Information:**

The online version contains supplementary material available at 10.1007/s12012-023-09802-9.

## Background and Aims

The electronic cigarette (EC) was introduced in 2003 as an alternative to traditional cigarette smoking. Over the years, the use of ECs has steadily increased across the world, especially among adolescents. This is partially due to major marketing efforts by the EC industry and e-liquid flavours especially targeting youth [1]. In Europe, 34% of 14–18-year-olds had tried ECs at least once in a 2017 survey [2]. The EC is composed of a battery-powered device, a resistance coil and a tank, filled with liquid that is aerosolised during inhalation. The liquid (e-liquid) consists of a mixture of propylene glycol and glycerol and, in most cases, nicotine as well as various flavourings. Inhalation of EC aerosol, often referred to as vaping, has been proposed as a tool for smoking cessation as well as for recreational use [3, 4].

Studies targeting EC-related health effects have demonstrated increased airway inflammation and obstruction, endothelial dysfunction, increased arterial stiffness and oxidative stress following EC use with e-liquids containing nicotine [5–8]. EC use exposes humans to several compounds with adverse effects on human health, such as fine particular matter, nicotine and volatile organic species [9]. Our group has previously demonstrated that brief exposure to EC aerosol containing nicotine results in increased levels of circulating endothelial progenitor cells (EPCs) as well as extracellular vesicles of endothelial and platelet origin, as a sign of vascular inflammation and endothelial activation [10–12].

The aim of the present study was to further investigate the impact of EC aerosol on vascular health applying well-established methods for assessing vascular function and haemostasis. Haemostasis was investigated by the Total-Thrombus-formation analysis system (T-TAS), which has been used for bedside assessment of thrombus formation in whole blood, evaluate anti-platelet therapies, anticoagulants and risk of bleeding during catheter interventions [13–16]. Microvascular function was measured with laser speckle contrast imaging (LSCI) and iontophoresis [17], a non-invasive optical laser technique that allows real-time assessment of skin microvascular reactivity. LSCI combined with iontophoresis to assess skin microcirculatory reactivity through endothelial-dependent and non-endothelial-dependent pathways has been used to assess endothelial dysfunction in patients with cardiac and metabolic diseases [18–20]. In patients with type 1 diabetes, reduced skin microvascular reactivity was associated with clinical microangiopathy, indicating that it may be a suitable model to assess general microvascular function [18–21]. We hypothesised that inhalation of EC aerosol would have detrimental effects on vascular function and haemostasis.

## Materials and Methods

### Study Design and Sample

Twenty-two healthy male and female occasional smokers or Swedish snus users (maximum 10 cigarettes or 10 pouches of snus per month) between 18 and 45 years of age were included in the study. Written informed consent was obtained at inclusion. The study design adhered to the 1975 Helsinki declaration and was approved by the Swedish Ethical Review Authority. The study was performed with a randomised, double-blind crossover design (Fig. 3, supplements). No significant carry-over effects were expected with a wash-out period of one week to allow for elimination of any inhaled nicotine. EC exposures, with and without nicotine, were performed on two occasions separated by a wash-out period of at least one week. Volunteers had to refrain from all sorts of nicotine products, water-pipe, drugs including marijuana, anti-inflammatory medications, and strenuous physical activity one week prior to exposures. Volunteers also had to abstain from caffeine and alcohol 24 h prior to exposure. Exclusion criteria were any chronic disease, infection, pregnancy, or inflammatory symptoms within the last 7 days prior to participation, and BMI below 18 kg/m^2^ or above 30 kg/m^2^. The study was started in the morning following an 8-h fasting period, and investigations were performed after 15 min of rest at a room temperature of 21–23 °C. Volunteers underwent simple randomisation after inclusion by the study supervisor. The two different E-liquids used for exposure were stored in identical containers marked with two different letters and content blinded to both parties.

All exposures were supervised and performed in a well-ventilated and temperature-controlled room. On each occasion, subjects inhaled one puff of EC aerosol per minute for 30 min. Each puff lasted 2–3 s. A third-generation EC (eVic Primo SE, Joyetech Electronics Co.,Ltd, China) was used, with a proC1-S atomizer with a resistance of 0.25Ω. Exposure settings were standardised (effect 32W, temperature 230 °C). Two commercially available e-liquids were used (Valeo laboratories GmbH, Germany), composed of propylene glycol (49%), glycerine (44%) and ethanol (5%) with no added flavourings; one contained 19 mg/ml of nicotine, the other without nicotine.

Venous blood was sampled from the antecubital vein with no or minimal stasis. At inclusion routine blood tests were taken including blood cell count, serum-creatinine, electrolytes and glucose. Blood pressure and pulse were measured at baseline, as well as at 30 and 60 minutes post exposure. Blood pressure equipment was a semi-automatic oscillometric sphygmomanometer (Omron M7, Omron Healthcare Europe B.V., Hoofddorp, NL).

### T-TAS

Total-Thrombus-formation analysis system (T-TAS®. Fujimoro Kogyo Co., Ltd., Japan) evaluates thrombus formation in whole blood using a microchip flow chamber system that can simulate various venous or arterial blood flow conditions [13]. T-TAS was evaluated at baseline and at 15 and 60 min post exposure. Two different microchips were used to evaluate thrombogenicity. The platelet chip (PL chip) consists of 25 artificial capillaries lined with type-1 collagen to evaluate platelet thrombus formation and primary haemostasis [22]. The atheroma chip (AR chip) consists of one single capillary lined with collagen and tissue factor that stimulates primarily fibrin-rich thrombus formation and activation of coagulation factors. All measurements in the study were made in accordance with the recommendations from the manufacturer.

Different shear rates simulate different flow conditions. For the PL chip, a flow rate of 18μL/min was chosen, corresponding to a shear rate of 1500 s^−1^, which represents conditions in arterioles. Blood was sampled in a BAPA canister (benzylsulfonyl-D-Arg-Pro-4-amidino benzylamide) for PL chip analysis. For the AR chip, we used a flow rate of 10μL/min, shear rate of 600 s^−1^, representing conditions in large arteries. For AR chip analysis, citrated whole blood was recalcified and treated with corn-trypsin inhibitor. Blood samples were processed 1 h following sampling. Analysis was made at a temperature of 36 °C. Analysis was carried out until near occlusion pressure was reached in the PL chip and AR chip capillaries. The pressure built up in the microchamber system following microthrombi formation was recorded on a graph. The recorded variables were T10 (the time in seconds to reach 10 kPa) and occlusion time (OT, in seconds, to reach occlusion)). Area under the curve (AR-AUC, PL-AUC) was calculated as an estimation of total thrombogenicity in the system. AR-AUC is a measurement of the area under the curve for 30 min after start of perfusion in the AR-chip, whereas PL-AUC is a measurement of the area under the curve for 10 min after start of perfusion in the PL chip. Increased thrombus formation in the capillaries cause earlier onset of T10, OT and an increase in AUC.

### Laser Speckle Contrast Imaging and Iontophoresis

Skin microvascular function was evaluated through investigation of changes in skin perfusion, assessed by laser speckle contrast imaging (LSCI) in response to iontophoresis of vasoactive drugs at baseline and 30 min after exposure [17]. LSCI (PeriCam PSI NR; Perimed, Järfälla, Sweden) is an optical technique that measures superficial skin flux by detection of red blood cell movements. The examined body part is kept stationary during the procedure and the method assessed skin perfusion in arbitrary units. Iontophoresis is a non-invasive method of applying drugs transdermally by using a small electrical current on the skin. Acetylcholine (ACh, Sigma-Aldrich AB, Stockholm, Sweden) and sodium nitroprusside (SNP, Hospira, Inc., Lake Forest, IL, USA), diluted in 0.9% sodium chloride solution to a final concentration of 2%, were used to evaluate endothelium-dependent and endothelium-independent microvascular function, respectively. On the volar side of the lower left arm, electrode chambers (LI611 Drug Delivery Electrode, Perimed, Järfälla, Sweden) filled with either ACh or SNP were attached. Drug iontophoresis was mediated at a current of 0.02 mA for 200 s. Skin microcirculatory perfusion was recorded before, during and after iontophoresis. Results were reported in perfusion arbitrary units (AU) providing values for basal skin perfusion, which was calculated as the mean value measured during 60 s before start of iontophoresis, and peak microcirculatory blood perfusion in response to ACh and SNP, respectively.

### Skin Temperature

Skin temperature of the volar side of left forearm and distal phalange of the left fourth digit was recorded with an electronic thermistor (Exacon, Copenhagen, Denmark) at baseline and 30 min following exposure.

### Statistical Analysis

It was planned to include 22 subjects due to the risk of drop-out and technical problems. Statistical analysis was performed in SPSS 27.0.0.0 64-bit edition (IBM Corporation, NY, US) and GraphPad Prism 8.4.2. (GraphPaSoftware Inc., CA, US). All data sets were tested for normality with Shapiro–Wilk test. Normally distributed data were expressed as means with standard deviations, while significantly skewed data were reported as medians and interquartile range. For normally distributed data, repeated measures ANOVAs and post hoc analysis with pairwise comparisons with Bonferroni correction were used to detect differences between groups. For skewed data, Friedman’s test together with post hoc analysis through Dunn’s pairwise comparisons and Wilcoxon signed-rank test were applied. P-values of < 0.05 were considered statistically significant. Any missing data point at any measurement point excluded that participant from the specific data analysis.

## Results

Twenty-two healthy individuals, 15 females and 7 males, were included in the study and analysis. Data collection took place between September 2019 and January 2020. Mean age was 27 ± 7, BMI was 24 kg/m^2^ ± 3 and waist circumference 79 cm ± 9. Baseline laboratory measurements showed mean haemoglobin of 134 g/L ± 14, white blood cell count 6 × 10^9^/L ± 2, platelet count 250 × 10^9^/L ± 51 and plasma-creatinine of 70 µmol/L ± 12.

### Skin Temperature, Heart Rate and Blood Pressure

Forearm skin temperature decreased significantly after exposure in both groups (Table 1) and no significant difference was detected between them. Similarly, finger skin temperature also decreased significantly after exposure to EC with and without nicotine. Heart rate increased significantly 30 min post exposure to EC aerosol with nicotine and was still altered at 60 min after completed exposure. In contrast, heart rate decreased significantly in the non-nicotine group. Systolic blood pressure was significantly elevated 30 min after EC exposure with nicotine compared with baseline (+ 9.25 mmHg, 95% CI: + 1.442 to 17.058; *p* = 0.17).

**Table 1:** Temperature and circulatory results before and after exposure to electronic cigarette aerosol with and without nicotine

Variable	Exposure	Baseline	Post exposure 30 min	Post exposure 60 min	*p* value
Forearm skin temperature	Nicotine Non-nicotine	30.1 ± 1.4 30.2 ± 1.1	29.2 ± 1.2 29.4 ± 1.2	NA	0.861
Finger skin temperature	Nicotine Non-nicotine	28.0 (5.8) 29.1 (5.0)	24.4 (2.8) 25.2 (2.9)	NA	** < 0.001**
Heart rate	Nicotine Non-nicotine	66 ± 10 68 ± 10	73 ± 13 63 ± 9	71 ± 13 64 ± 11	** < 0.001**
Systolic blood pressure	Nicotine Non-nicotine	108 ± 14 110 ± 11	117 ± 14 113 ± 13	112 ± 10 114 ± 13	0.067
Diastolic blood pressure	Nicotine Non-nicotine	70 ± 12 70 ± 9	74 ± 8 73 ± 8	74 ± 7 75 ± 9	0.928

Effects of exposure to electronic cigarette aerosol with and without nicotine on forearm skin temperature, finger temperature, heart rate, systolic and diastolic blood pressure

Parametric data are presented as mean values ± standard deviations. Non-parametric data presented as median (interquartile range). P-values are repeated measures ANOVAs or Friedman’s test, Exposure x Time

Bold values indicates the P-values below 0.05 was considered statistically significant

### T-TAS

**Fig. 1:**
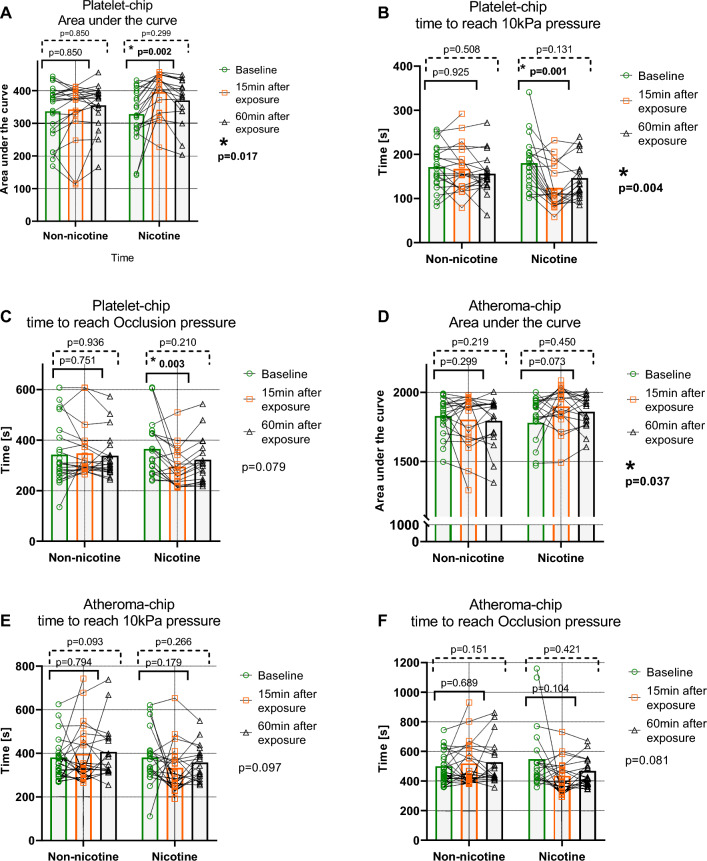
Platelet and fibrin-rich thrombus formation following exposure to electronic cigarette aerosol with and without nicotine. Graphs show individual values and bars median values. P-values for Friedman’s test (exposure and time) are shown on the right side. P-values above the brackets represent post hoc analysis with pairwise comparisons baseline vs post exposure

T-TAS measurement data are presented in Fig. 1 and table 2 (supplements). In brief, PL-AUC increased and PL-T10 decreased significantly after 15 min following exposure to EC with nicotine (Fig. 1a–b). PL-OT showed a trend of decreasing following exposure to EC with nicotine (Fig. 1c). However, post hoc analysis of PL-OT detected a significantly faster platelet thrombus occlusion time after 15 min following exposure both compared to non-nicotine exposure and baseline (*p* = 0.030 and *p* = 0.003, respectively, Wilcoxon’s signed-rank test), altogether indicating increased platelet thrombogenicity under flow.

AR-AUC increased significantly following EC exposure with nicotine (Fig. 1d). Both AR-T10 and AR-OT showed a trend towards a decrease following EC exposure with nicotine after 15 min and partially returned to baseline after 60 min (Fig. 1e–f). AR-T10 following nicotine exposure was reached significantly faster compared with the non-nicotine exposure after 15 and 60 min (*p* = 0.038 and *p* = 0.044, respectively, Wilcoxon’s signed-rank test). Similarly, AR-OT was reached significantly faster following nicotine exposure compared with non-nicotine exposure both at 15 and 60 min (Fig. 1f *p*= 0.010 and *p* = 0.049, respectively, Wilcoxon’s signed-rank test). Thus, also fibrin-dependent thrombogenicity increased following nicotine exposure.

### LSCI

**Fig. 2:**
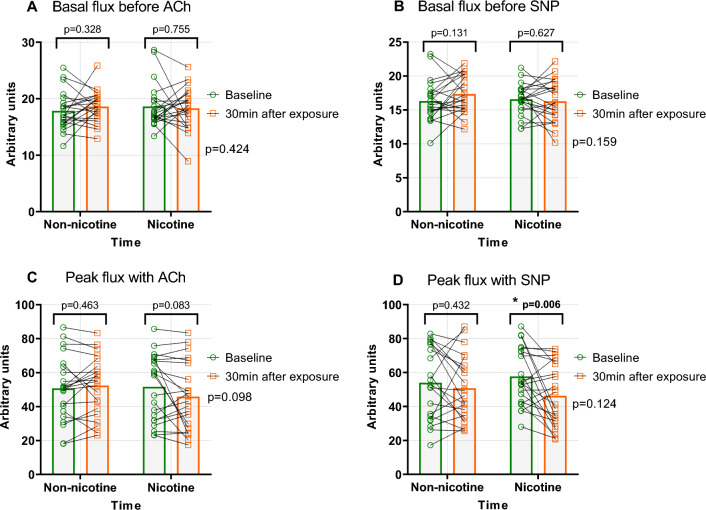
Endothelial-dependent and independent skin microcirculation following exposure to electronic cigarette aerosol with and without nicotine. Basal skin flux before iontophoresis of ACh and SNP^a, b^ and skin microvascular reactivity in response to ACh^c^ and SNP^d^ before and after exposure to either nicotine or non-nicotine electronic cigarette aerosol. Graphs display individual values and bars mean values. P-values for repeated measures ANOVAs, (Exposure x Time) are presented on the right side of the graphs. P-values above the brackets represent post hoc analysis with pairwise comparisons baseline vs post exposure. *ACh* Acetylcholine, *SNP* Sodium nitroprusside

LSCI data showing changes in skin microvascular reactivity following EC exposure are presented in Fig. 2 and table 3 (supplements). One subject was excluded from SNP provocation due to technical failure. In summary, following EC exposure, no significant difference in SNP or ACh-mediated peak perfusion was found between the nicotine and non-nicotine group. However, post hoc analysis with pairwise comparisons showed that SNP-mediated peak perfusion was significantly reduced following nicotine EC exposure compared with baseline (− 12.4AU, 95% CI: − 20.8 to − 4.0; *p* = 0.006). For ACh-mediated peak perfusion a decreasing trend was seen following nicotine exposure compared with baseline in the post hoc analysis (− 5.7AU, 95% CI: − 12.3 to − 0.8; *p* = 0.083). Resting skin perfusion levels were not altered following either exposure.

## Discussion

We investigated the effects of brief EC inhalation with and without nicotine on platelet function and coagulation and microvascular function in healthy individuals. We demonstrate that exposure to EC aerosol containing nicotine was associated with increased thrombogenicity in terms of increased platelet thrombus formation and fibrin-rich thrombus formation in whole blood under flow conditions. Both the nicotine and non-nicotine EC aerosol exposures were associated with altered skin perfusion, displayed by reduced forearm and digit skin temperatures. Furthermore, inhalation of EC aerosol with nicotine had an acute impact on macro- and microvasculature in terms of increased heart rate, elevated systolic blood pressure and reduced endothelial-independent (SNP-mediated) vasodilation.

Baseline T-TAS data for subjects prior to exposure correlated to other studies employing healthy controls at a similar age range [23]. Our results demonstrate an increase in both platelet thrombus formation (PL-chip) and an increased platelet-dependent coagulation activation tendency (AR-chip) following EC exposure with nicotine. Of note, the T-TAS AR-chip is coated with both collagen and tissue factor, thus also including activation of coagulation and fibrin formation in addition to the platelet dependency assessed with the PL-chip, where a thrombin inhibitor is added in the system. Hence, our data indicate that EC exposure with nicotine also enhance fibrin-dependent thrombus formation possibly leading to a firmer thrombus.

The present result of increased platelet thrombus formation following EC nicotine exposure is in line with the previous studies that have demonstrated increased platelet aggregation, heightened levels of extracellular vesicles of platelet origin and increased amounts of P-selectin in blood following EC use with nicotine in healthy individuals [10, 24, 25]. Compared with these studies, the application of the T-TAS to study platelet reactivity and function has the advantage that it evaluates platelet function under physiological flow conditions [26]. The T-TAS PL and AR chip measurements incorporate multiple pathways of platelet and coagulation activation, including rheological effects, activation of coagulation factors, release of endogenous platelet agonists, and vWF-mediated platelet adhesion and platelet aggregation [13]. There have been suggested reference intervals for the PL chip and inter-individual variability is high making it suitable for crossover studies such as the current one [27]. Furthermore, the method has been shown to be sensitive to detect traditional risk factors for cardiovascular disease [27].

A potential mechanism for EC aerosol with nicotine to induce the effects demonstrated is through the release of catecholamines that activate platelets and the sympathetic nervous system. A brief catecholamine surge caused by EC nicotine use may explain the temporary increase in thrombogenicity, increased heart rate, blood pressure and reduced skin temperature [28–30]. Nicotine releases endogenous norepinephrine and epinephrine, which may activate platelets through binding to alpha-2 adrenergic receptors on platelets [29, 31–33]. Furthermore, exposure to traditional cigarettes causes increased platelet aggregation and thrombin generation in humans [29, 34, 35]. It has been proposed that the demonstrated effects of nicotine in humans are dose-dependent [36]. The effects of nicotine on platelet activation also relate to the delivery system used, with one study indicating that transdermal patches result in less platelet activation than conventional cigarettes [37]. In contrast to steady-state slow-release systems, bolus-type delivery system such as inhalation may trigger more detrimental cardiovascular and thrombotic responses [38, 39]. It has been shown that ECs deliver nicotine to users at least as efficiently as conventional cigarette smoking [40]. As the findings in the present study only showed thrombogenesis and thrombus formation following use of ECs containing nicotine, this might further support nicotine as the main underlying cause of this adverse effect.

There are several other constituents in the EC aerosol besides nicotine that have previously been suggested to affect thrombogenesis, including volatile organic compounds, ethanol and ultrafine particles [41, 42]. In the present study, no effects on thrombogenicity were observed following exposure to EC without nicotine. Possibly, this is due to our small sample size and comparably short exposure to EC aerosol.

Following EC exposure with nicotine, heart rate increased significantly and systolic blood pressure showed trend of increasing compared with EC exposure without nicotine. This effect is possibly mediated by nicotine, inducing the release of neurotransmitters such as norepinephrine activating the sympathetic nervous system [43]. The observed result of increased heart rate and blood pressure following EC exposure with nicotine is in line with other studies [44]. In addition, we observed lowered basal skin perfusion measured through skin temperature on the forearm and finger, following both nicotine free and nicotine EC exposures which indicates an effect driven by other components than nicotine. It is well established that both conventional cigarette smoking and nicotine cause peripheral vasoconstriction and, consequently, impaired wound healing following surgery [45]. Besides a small pilot study, this is the first study evaluating effects of EC use on skin temperature [46]. Lowered skin perfusion following nicotine-free EC exposure could be due to other compounds in the EC, such as fine particulate matter, that has previously been suggested to increase sympathetic activity [47]. In the skin, different vasoconstrictor and vasodilator sympathetic nerves regulate the cutaneous blood flow in order to maintain thermoregulation [48]. At rest and thermoneutral conditions, the skin microcirculation is mainly influenced by vasoconstrictors and the blood flow is low. Decreased skin temperature, observed during EC exposure, might indicate an increased vasoconstrictor sympathetic activity. However, more research focussing on other compounds in the EC aerosol is needed to establish the exact cause of this finding.

No significant difference was detected between the two exposures when evaluating either endothelial-independent microcirculation (SNP-mediated response) or endothelial-dependent (ACh-mediated) microvascular response. However, nicotine containing EC exposure led to a significantly decreased SNP-mediated microvascular reactivity compared with baseline values, indicating reduced vasodilation capacity by the smooth muscle cells. SNP is a pro-drug that releases nitric oxide (NO), which is a potent vasodilating compound that diffuses into the vascular smooth muscle cells where it activates soluble guanylyl cyclase and causes vasorelaxation through formation of cyclic guanosine monophosphate (cGMP). ACh, on the other hand, exerts its effect on the vascular endothelial cells and stimulates these cells to release various vasodilating compounds including NO, prostanoids and endothelial-derived hyperpolarising factors which in turn act on the smooth muscle cell and cause them to relax. A reduction in mainly SNP-mediated vasodilatation could be explained by reduced NO bioavailability, as nicotine exposure is associated with increased oxidative stress and reactive oxygen species and free radicals react with NO [49, 50]. Animal studies have also shown a correlation between increased catecholamine levels and reduced NO levels [51]. In humans, chronic EC users have a reduced vessel dilatation evaluated as flow-mediated dilation (FMD) in the brachial artery [52]. Similarly short-term EC exposure studies with and without nicotine in healthy young adults have demonstrated reduced vessel dilatation in FMD following exposure [24, 53, 54]. While FMD reflects vascular reactivity in a larger artery following arterial occlusion and increased shear stress on the vascular endothelial cells, our study investigated vascular reactivity in the microcirculation following pharmacological drugs that act on endothelial cells and smooth muscle cells, respectively. Altogether, the results of our project and previous studies on vascular function suggest that EC and nicotine exposure reduces the vascular dilatation capacity in both the micro- and macrocirculation through yet unknown mechanisms that seem to involve both endothelial cell and vascular smooth muscle cell function.

## Limitations and Strengths

The main strength of this study was the randomised double-blind crossover design that reduced bias and number of participants needed. The study included a disproportional number of females compared with males and participants were healthy and relatively young individuals, which makes it hard to extrapolate results to older individuals with established vascular disease. Present results might not be applicable to regular EC users, as the exposure regime of 30 puffs in this study is low compared with the mean vaping amount of 120–235 puffs daily estimated for regular EC users [55]. One limitation of the study is that the sample size was too small for investigation of skin microvascular reactivity, and the negative data in these analyses could be due to type II error. The T-TAS method is limited as it does not provide detailed information of any detected platelet or coagulation dysfunction, making it suitable for evaluation of general haemostasis only. Furthermore, most previous studies using the T-TAS method have evaluated bleeding complications and there are no clearly identified threshold values associated with thrombotic risks [15, 16].

## Conclusion

This study shows that a brief exposure to EC aerosol containing nicotine acutely increased thrombus formation and possibly reduced endothelium-independent microvascular reactivity. These changes were not seen after EC vaping without nicotine, which implies that the potential harmful effects of EC seen in the present study on thrombogenicity and microvascular function may be mediated through nicotine.

## Supplementary Information

Below is the link to the electronic supplementary material.

Supplementary file1 (DOCX 113 KB)

## Data Availability

The data collected for the study can be made available to others upon request and after a confidentiality evaluation.
