# Supplementary material for: Electronic Cigarette Vaping with Nicotine Causes Increased Thrombogenicity and Impaired Microvascular Function in Healthy Volunteers: A Randomised Clinical Trial
Source: Cardiovasc Toxicol. 2023 Aug 7;23(7-8):255–64. doi: 10.1007/s12012-023-09802-9 (PMC10435650; doi:10.1007/s12012-023-09802-9)
Supplement: Supplementary file 1 — Supplementary file1 (DOCX 113 KB) [file 12012_2023_9802_MOESM1_ESM.docx]

**Supplementary material:**

**Figure 3. Flowchart of randomised crossover design.**

**
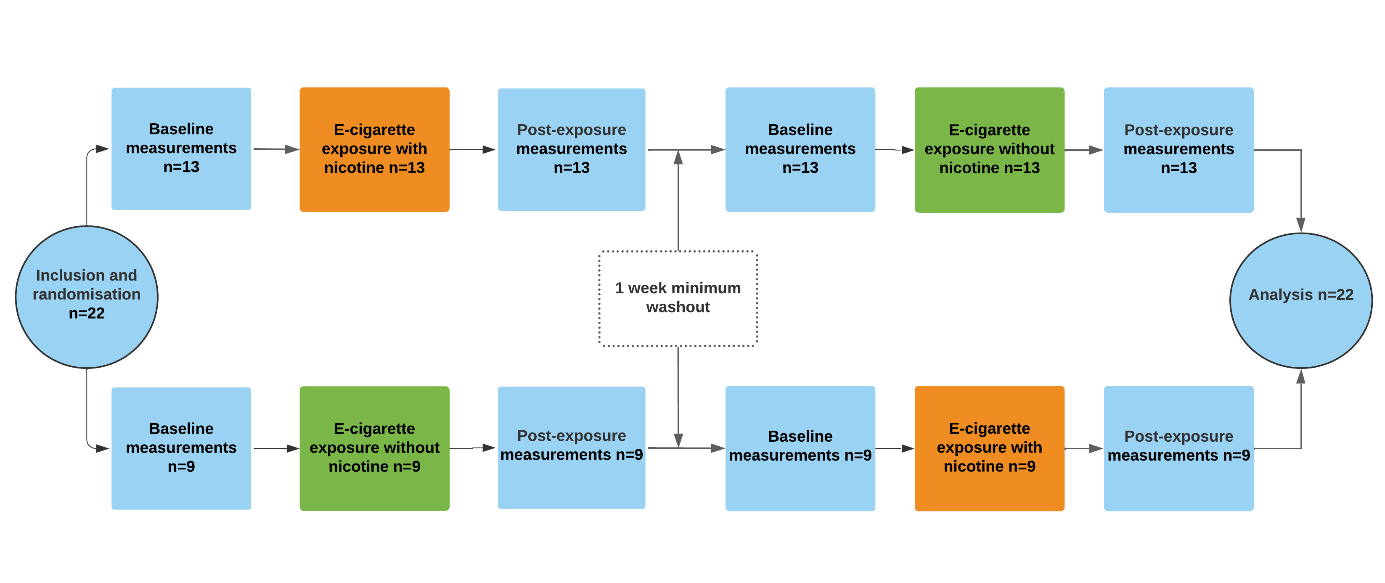
**

**Table 2. T-TAS results, displaying changes in thrombotic following nicotine and non-nicotine electronic cigarette exposure.**

| **Variable** | | **Baseline** | **15 min after exposure** | **Nicotine vs non-nicotine**  **p-value** | **60 min after exposure** | **Nicotine vs non-nicotine**  **p-value** | **Friedmans test**  **p-value** |
| --- | --- | --- | --- | --- | --- | --- | --- |
| AR-T10 (s) | Nicotine  Non-nicotine | 373 (111–621) [21]  378 (269–625) [22] | 309 (191–653) [20]  334 (264-743) [21] | **0.038** | 343 (254-549) [17]  367 (255-738) [18] | **0.044** | 0.097 |
| AR-OT (s) | Nicotine  Non-nicotine | 470 (358-1160) [21]  486 (359-744) [22] | 412 (293-731) [20]  445 (379-930) [21] | **0.010** | 423 (345-670) [17]  464 (356-861) [18] | **0.049** | 0.081 |
| AR-AUC | Nicotine  Non-nicotine | 1834 (1040-2002) [21]  1844 (1498-1992) [22] | 1933 (1492-2086) [20]  1897 (1293-1977) [21] | **0.006** | 1902 (1605-2009) [17]  1864 (1347-2004) [18] | 0.059 | **0.037** |
| PL-T10 (s) | Nicotine  Non-nicotine | 176 (101-341) [20]  171 (83-256) [22] | 108 (58-232) [19]  156 (79-292) [21] | **0.003** | 133 (85-240) [17]  148 (62-272) [19] | 0.073 | **0.004** |
| PL-OT (s) | Nicotine  Non-nicotine | 342 (240-609) [20]  308 (135-608) [22] | 272 (212-510) [19]  296 (265-608) [21] | **0.030** | 307 (218-543) [17]  306 (244-573) [19] | 0.352 | 0.079 |
| PL-AUC | Nicotine  Non-nicotine | 337 (141-432) [20]  369 (169-444) [22] | 416 (226-457) [19]  373 (112-413) [21] | **0.003** | 385 (203-448) [17]  379 (165-455) [19] | **0.345** | **0.017** |

Non-normally distributed variables expressed as median (range) [n]. P-value represents Friedman's test as omnibus test and post hoc analysis with pairwise comparisons between exposure groups at the specific time point. AR: Atheroma-chip; PL: Platelet-chip; T10: Time to reach 10kPa pressure in microchip; OT: Time to reach occlusion pressure in microchip; N: Number; df: degrees of freedom.

**Table 3. Laser speckle contrast imaging with iontophoresis displaying changes in microcirculatory flux following exposure to either nicotine or non-nicotine electronic cigarette exposure.**

| **Variable** | | **Baseline** | **30min after exposure** | **Baseline**  **vs**  **postexposure**  **p-value** | **Postexposure vs postexposure**  **p-value** | **Repeated measures ANOVA**  **p-value** |
| --- | --- | --- | --- | --- | --- | --- |
| Basal flux before ACh (AU) | Nicotine  Non-nicotine | 18.6 ± 3.9  17.8 ± 3.4 | 18.3 ± 3.7  18.6 ± 2.8 | 0.755  0.328 | 0.754 | 0.424 |
| Peak flux with ACh (AU) | Nicotine  Non-nicotine | 51.6 ± 19.3  50.8 ± 19.0 | 45.9 ± 20.0  52.4 ± 17.2 | 0.083  0.463 | 0.133 | 0.098 |
| Basal flux before SNP (AU) | Nicotine  Non-nicotine | 16.6 ± 2.3  16.3 ± 3.0 | 16.3 ± 3.1  17.3 ± 2.7 | 0.627  0.131 | 0.197 | 0.159 |
| Peak flux with SNP (AU) | Nicotine  Non-nicotine | 57.7 ± 16.8  54.0 ± 21.3 | 46.3 ± 18.1  50.7 ± 18.9 | **0.006 ***  0.432 | 0.312 | 0.124 |

Normally distributed values expressed as mean ± SD. N=22 for all measurements. P-values are repeated measures ANOVAs and post-hoc analysis with pairwise comparisons and Bonferroni correction. AU: Arbitrary units; ACh: Acetylcholine; SNP: Sodium nitroprusside.
